# Effect of Hope-oriented group counseling on mental health of infertile women with failed IVF cycles: a randomized controlled trial

**DOI:** 10.1186/s12888-021-03280-5

**Published:** 2021-06-02

**Authors:** Roya Rahimi, Shirin Hasanpour, Mojgan. Mirghafourvand, Khalil Esmaeilpour

**Affiliations:** 1 Student Research Committee, Department of Midwifery, Nursing and Midwifery Faculty, Tabriz University of Medical Sciences, Tabriz, Iran; 2 Women’s Reproductive Health Research Center, Tabriz University of Medical Sciences, Artesh Street, Tabriz, Iran; 3 Midwifery Department, Social Determinants of Health Research Center, Tabriz University of Medical Sciences, Tabriz, Iran; 4 Faculty of Education and Psychology, University of Tabriz, Tabriz, Iran

**Keywords:** Infertility, IVF, Mental health, Counseling, Hope therapy

## Abstract

**Background:**

Considering the prevalence of infertility in the community and the consequences of failure of infertility treatments on women’s mental health, interventions that can control stress, anxiety and depression in infertile women with a history of IVF failure will be very helpful. This study aimed to determine the effects of hope-oriented group counseling on mental health (primary outcome) and quality of life (QoL) (secondary outcome) of women with failed IVF cycles.

**Method:**

This randomized controlled trial was conducted on 60 women with failed IVF cycles visiting Infertility Clinic at Al-Zahra Teaching Hospital of Tabriz- Iran. Participants were allocated to the intervention group (*n* = 30) and control group (*n =* 30) based on a randomized block design. Hope-oriented group counseling was provided to the intervention group in six 45–60 min sessions (once a week). The control group only received routine care to undergo another IVF cycle. The Depression Anxiety Stress Scale-21 (DASS-21) and the SF-12 Quality of Life Scale were filled out by interviewing the participants before the intervention and one week and one month after the intervention. After intervention 26 participants in each group were included in the analysis.

**Results:**

There was no significant difference between the intervention and control groups in the socio-demographic profile of participants (*P* > 0.05). The post-intervention mean score of stress (adjusted mean difference = − 1.7, 95% confidence interval: − 3.2 to − 0.3, *P* = 0.018) and depression (adjusted mean difference = − 1.3, 95% confidence interval: − 4.7 to − 1.5, *P* < 0.001) was significantly lower in the intervention group compared to the control. Although the mean anxiety score was lower in the intervention group compared to the control, the difference between them was not statistically significant (adjusted mean difference = − 1.1, 95% confidence interval: − 2.6 to 0.4, *P* = 0.153). The mean score of QoL was significantly higher in the intervention group than that of the control group (adjusted mean difference = 6.9, 95% confidence interval: 5.1 to 8.8, *P* < 0.001).

**Conclusion:**

Hope-oriented group counseling was effective in reducing stress and depression and improving QoL in women with failed IVF cycles. It is recommended to use this counseling approach, along with other methods, to improve the mental health of women with failed IVF cycles.

**Trial registration:**

TCT Registration Number: TCTR 20191017003, registered on October 17, 2019.

## Introduction

Infertility is defined as the inability of a couple to conceive after 1 year of continuous sexual contact without the use of contraception [1].. According to studies, about 11 to 51 million people suffer from a type of infertility worldwide in such a way that one out of six couples of reproductive age has this problem [2]. As shown in the literature, the mean global infertility rate is 10% [3]. Parsanejad et al. (2016) showed that the total infertility rate in Iran was 10.9% [4]. When the desire to have children is unfulfilled, couples face a mutual ambiguity; they psychologically desire to have children but it is physically impossible for them. This ambiguity and confusion impose huge stress on people [5]. Infertility-specific stress refers to a group of symptoms that appear following infertility diagnosis; these symptoms are similar to those of post-traumatic stress disorder and are specifically evident in the beliefs and feelings associated with infertility [6].

Various infertility treatments, from medical and hormonal examinations to fertility techniques such as ART (Assisted Reproductive Technology), are stressful for infertile women and impose a substantial mental and physical burden on women and their spouses [7]. Prolonged exposure to high levels of stress leads to depression and anxiety, which are considered a threat to IVF/ICSI (In Vitro Fertilization/ Intra Cytoplasmic Sperm Injection) treatment outcomes [8].

Considerable attention has been recently paid to the role of psychological factors in infertility, and medicine suggests a relationship between infertility and psychological factors [9]. Although psychological factors are not the main cause of infertility, studies indicate that they may be involved in infertility [10]; the higher the rate of treatment failure, the more the burden of psychological pressure on women [11]. The overall prevalence of mental disorders in infertile couples has been reported to range between 25 and 60% [9].

Infertility-specific stress affects all aspects of quality of life (QoL) and life satisfaction of infertile couples [12]. The adversity of infertility, prolonged treatments, frequent visits and follow-ups, and the high costs of infertility treatments affect various aspects of QoL, especially emotional and social aspects. On the other hand, poor QoL contributes to the reduced chance of IVF success [13]. Researchers suggest that infertility treatment plans should be combined with a psychotherapeutic treatment plan [14]. Counseling and referral for more advanced psychological diagnosis and treatment could reduce maternal and neonatal morbidity and mortality rate, in addition to improving QoL [15].

While diagnosing the infertility-cause stresses and damages, psychological interventions help infertile couples work through their problems and adapt to infertility [16]. Several studies showed that socio-psychological interventions for couples undergoing infertility treatments can effectively reduce mental health problems and improve clinical pregnancy rate [17, 18]. Cognitive-behavioral therapy is a highly efficient short-term treatment in psychology that focuses on modifying dysfunctional behaviors and thoughts to help individuals experience general mental health [19]. Hope therapy is one of these psychological interventions. Hope is a physiological need of humans that brings them flexibility, freshness, and the ability to get rid of life harms and also improves their mental health [20]. Considering the effects of hope-promoting group interventions and interaction-induced insight and learning, hope-oriented group counseling can play a major role in improving QoL of patients with incurable and chronic diseases [21].

Studies about the effects of hope-oriented group counseling on the mental health status of infertile women indicated that hope therapy, as a positive approach, can improve the general mental health of infertile women and, consequently, improve their family’s mental health, QoL, and adaptation to life problems [22]. A case study on women visiting public and private gynecological and obstetric medical centers in Ilam- Iran in 2013 demonstrated that the physical and mental QoL of infertile women was lower than that of fertile women. Therefore, it is essential to apply appropriate counseling and necessary training to improve their QoL [23].

Some studies have shown that psychotherapeutic interventions improve mental health, reduce anxiety and depression, and increase the fertility rate. Researchers believe that psychological counseling is essential before and during IVF cycles and couples seek psychological counseling when undergoing IVF [24, 25]. The study by Sumer et al. indicated that couples, especially infertile women, lacked sufficient knowledge on infertility and its treatment methods [26]. According to the studies, in order to prevent the incidence and exacerbation of psychiatric disorders, researchers recommend counseling and psychotherapeutic interventions to help infertile couples [16, 25].

Given what was mentioned above, failed IVF cycles have negative effects on women’s mental health and their anxiety and stress can negatively affect IVF results. According to the results of some studies, counseling interventions promote the mental health of women undergoing IVF. In addition, few studies have investigated the effects of hope-oriented group counseling on the mental health of women with failed IVF cycles, either in Iran or other countries. This study hence aims to determine the effects of hope-oriented group counseling on mental health (primary outcome) and quality of life (secondary outcome) of infertile women with failed IVF cycles.

## Methods

### Research design and participants

This was a randomized controlled trial conducted on 60 women with failed IVF cycles visiting the Infertility Clinic of Al-Zahra Teaching Hospital of Tabriz. Sampling (recruitment and randomization) was conducted from September 2019 to January 2020, the intervention lasted for 6 weeks from January 2020 to mid-February 2020 and the follow-up lasted for 1 month after the intervention from mid-February 2020 to mid-March 2020. The inclusion criteria were women with failed IVF cycles, minimum educational attainment of junior high school, living in Tabriz, having a landline telephone number and a mobile phone number and getting a score of 8 or higher on the anxiety questions of DASS-21 scale. The exclusion criteria were the history of psychiatric problems, self-reported psychotropic medication use, self-reported addiction to drugs, cigarettes, and alcohol, self-reported history of chronic physical problems (cardiac disorders, hypertension, pulmonary diseases, iron deficiency anemia, diabetes, thyroid disorders, epilepsy), and self-reported severe psychological crisis during the last 3 months, such as the death of relatives.

According to the results of the study by Mosalanejad et al. [22] and the results of the DASS scale in infertile women, where m_1_ = 29.06 (DASS score before intervention), with a 25% presumed decrease in the mean DASS score following intervention (m_2_ = 21.795), SD_1_ = SD_2_ = 7.71, one-sided α = 0.05, and Power = 95%, a sample size of 26 was calculated in G*Power. Assuming an attrition rate of 15%, 60 participants were allocated to the control group (*n* = 30) and the intervention group (*n =* 30). A change of 4 in DASS was considered a minimally clinically important difference (MCID) [27]. Therefore, with this calculated sample size, the trial has 45% power to detect a difference of 4 with a two-sided test, and 58% with a one-sided test.

### Sampling and randomization

Sampling initiated after obtaining the ethical approval code from Ethics Committee of Tabriz University of Medical Sciences (IR.TBZMED.REC.1398.382) and registering the study information on the Registry Center of Clinical Trials (TCTR 20191017003). The author visited the Infertility Clinic of Tabriz Al-Zahra Teaching Hospital and obtained the list of infertile women undergoing IVF in the last 2 weeks (pregnancy test results are determined 2 weeks after IVF). Then they were called and, in case of a failed pregnancy, they were briefed on the research objectives and procedures. They were also examined for the inclusion and exclusion criteria. To encourage eligible women to participate in the study, the importance of psychotherapy and its role in improving the results of later treatments were explained and they were invited to attend the clinic if they were willing to participate in the study.

In a face-to-face meeting, the research objectives and methods were re-explained thoroughly to women and it was mentioned that if the counseling has a positive effect, this intervention will be done for the control group too. If they were willing to participate in the study, the researcher filled out the anxiety questions of DASS-21 Scale by interviewing them. Those who scored 8 and above entered the study after confirming their desire to participate in all counseling sessions and obtaining informed written consent. Then the socio-demographic information form, DASS-21 and SF-12 Quality of Life Scale were filled out by interviewing the participants.

Participants were equally allocated to the intervention (counseling) group and the control group using a randomized block design (blocks of 4 and 6, www.random.org) considering the number of failed IVF cycles (once and twice or more) and cause of infertility (male or female). In order to conceal the allocation, the type of intervention was written on paper and placed in numbered opaque envelopes by a person who was not involved in sampling and data analysis. Envelopes were opened in the order of participant recruitment and they were allocated to the intended group with 1:1 allocation ratio. In this study the data analyzer was blinded to the study groups.

### Intervention

Hope-oriented group counseling was provided to participants of intervention group (in groups of 7–8) in six 45–60-min sessions (once a week) and telephone follow-up was performed by the author in the interval between sessions to remind the next session and answer possible questions. In case of non-participation of any person in any session, she was invited to another group to hold the same session during the same week.

Counseling was provided by the author in vernacular language at Infertility Clinic of Al-Zahra Teaching Hospital of Tabriz in a room dedicated to counseling with a friendly and quiet environment. The content of the sessions was as follows:

#### First session

Welcoming and introduction, explaining the aim of the plan and intervention, establishing initial communication with clients, and encouraging them to express their feelings and thoughts through open-ended questions, active listening, and feedback on their concerns.

#### Second session

Providing information on hope and its positive outcomes and the role of hope in improving mental health and quality of life.

#### Third session

Organizing the components of hope and providing ways to achieve goals by participants. Participants were asked to write down their positive and negative feelings on infertility as their assignment and express them in the next session.

#### Fourth session

Reviewing the assignments of the previous session, promoting hope by explaining clear problem-solving methods and setting promising therapeutic goals, and inviting spouses to participate in the fifth session.

#### Fifth session

Maintaining hope by encouraging participants to think purposefully, identifying barriers and using mini interventions to maintain hope such as joining a hopeful person in their lives and visiting them to discuss current objectives and barriers, and reviewing personal hopeful sentences. The participants were asked to write down perceptual barriers to achieving their goals and some hopeful sentences as their assignment to present them in the next session.

#### Sixth session

Reviewing the assignments of the previous session, asking and answering questions, summing up, and reviewing what was taught in the previous session.

The author’s phone number was given to the participants to call in case of needing more counseling. The control group only received routine care. One week and one month after the intervention, the participants were called and interviewed by the researcher to fill out the post-intervention questionnaires. The person completing the questionnaires with interview was separate from the counselor and was blind to the study groups.

At the end of the study, hope therapy counseling was also performed for the control group.

### Data collection tools

Data were collected using the inclusion and exclusion checklists, a socio-demographic information form, DASS-21, and SF-12 Quality of Life Scale.

#### Socio-demographic information form

This form included questions on age, educational attainment, job, spouse’s age, spouse’s educational attainment, spouse’s job, duration and cause of infertility, number of children, number of pregnancies, number of abortions, number of stillbirths, number of IVF cycles, duration of infertility treatment, having a saved embryo, history of pregnancy through egg donation, level of family income adequacy for living expenses, place of residence, marital satisfaction, worrying about family relations in case of IVF failure, and stress of failure during the infertility treatment process. This was a researcher-made form whose content validity was confirmed by 10 faculty members at Tabriz University of Medical Sciences.

#### Depression, anxiety, stress scale- 21(DASS-21)

The Depression, Anxiety and Stress Scale-21 Items (DASS-21) is the short form of DASS-42 developed by Lovibond and Lovibond in 1995. It consisted of 21 items in three subscales of stress, depression, and anxiety with 7 questions apiece. The items were scored based on a Likert scale from NEVER (0) to VERY MUCH [3]. The score of each subscale is calculated separately but the total score is not calculated. Minimum and maximum scores for each subscale were 0 and 21, respectively [28] . Anthony et al. (1998) conducted factor analysis for this scale and their results also confirmed three factors of depression, anxiety, and stress. The results of this study suggested that 68% of the total variance of the scale was measured by these three factors. The eigenvalues of stress, depression, and anxiety in the study were 9.07, 2.89, and 1.23 with an alpha coefficient of 0.97, 0.92, and 0.95, respectively. In addition, the results of correlation between these factors in the study of Anthony et al. (1998) showed a correlation between depression and stress (r = 0.48), between anxiety and stress (r = 0.53), and between anxiety and depression (r = 0.28) [29]. The validity and reliability of this questionnaire were investigated in Iran by Samani and Jowkar (2007). The test-retest reliability for depression, anxiety, and stress were 0.81, 0.78, and 0.80, respectively, and Cronbach’s alpha for depression, anxiety, and stress were 0.81, 0.74, and 0.78 respectively [30].

#### Quality of life SF-12

The SF-12 Quality of Life Scale is the short form of the QoL-36 Scale that is extensively used in various studies [31] . The 12-item QoL Scale was developed by Ware et al. in 1996 and its reliability and validity were confirmed by Kamkari et al. in 2010 in Iran [32]. The validity of physical and mental items of this scale was calculated 0.67 and 0.97 respectively and the reliability of the scale was measured using the test-retest reliability and a correlation of 0.89 and 0.76 was reported for 12 items [33]. Given the low number of items, the total score of participants is mainly used. The present scale evaluates QoL in terms of the general perception of health (Item 1), physical function (items 2 and 3), physical health (items 4 and 5), physical problems (items 7 and 6), physical pain (Item 8), social functioning (Item 9), vitality and vital energy (Item 11) and mental health (items 10 and 12). The items were designed using both the Likert scale and YES/NO questions. The total score was calculated by summing up the scores of 12 items, which ranged between 0 and 36; higher scores indicated higher levels of QoL [32, 34].

The reliability of the DASS-21 and the SF-12 Quality of Life Scale was evaluated using the test-retest reliability with a two-week interval on 20 women with failed IVF cycles. Cronbach’s alpha (internal coherence) and intra-correlation coefficient (ICC) were calculated. The ICC and Cronbach’s alpha coefficient were 0.88 and 0.87 for the DASS-21 and 0.85 and 0.91 for the SF-12 Quality of Life Scale respectively.

### Statistical analysis

The data collected from all participants were analyzed in SPSS-24. The normality of quantitative data was evaluated using the Kolmogorov-Smirnov test. Descriptive statistics including mean and standard deviation, frequency and percentage were used to describe socio-demographic charactristics of both group participants and the chi-square test, trend chi-square test, Fisher’s exact test, and the independent t-test were used to investigate similarities between the groups in terms of socio-demographic profile. The independent t-test was used to compare baseline DASS and QoL Scales between the groups. Repeated measures ANOVA, controlling for baseline scores, was used to compare mean scores of DASS and QoL Scale between the two groups at follow-up. The overall adjusted mean difference is presented with a 95% confidence interval and *p*-value**.**

## Results

**Fig. 1:**
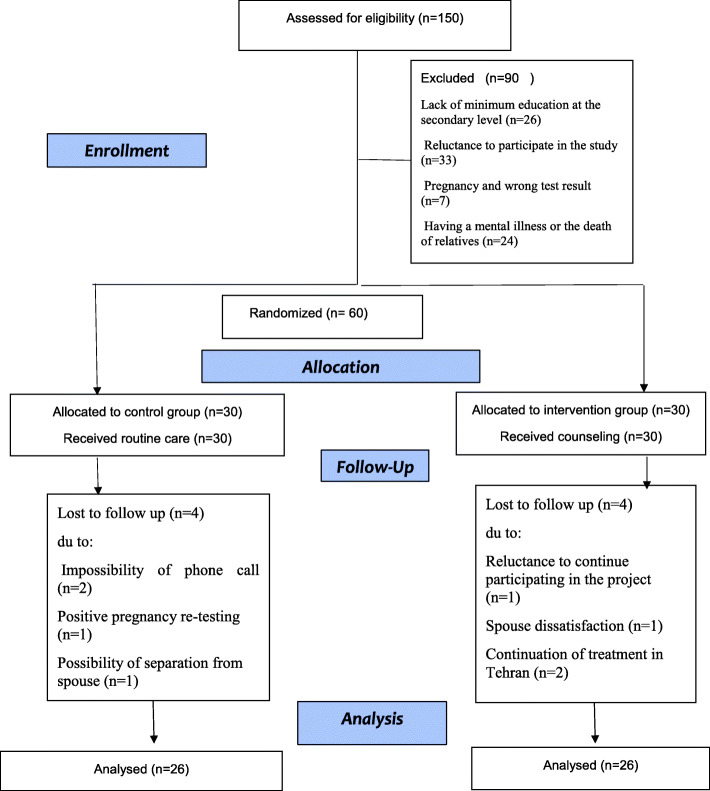
Flow Diagram

The study was conducted from September 2019 to January 2020. First, 150 women with a negative pregnancy test were called and 90 of them who did not meet the inclusion criteria were excluded from the study (26 women for education attainment lower than junior high school, 7 women for pregnancy and false-negative test result, 33 women for reluctance to participate in the study, and 24 women for having psychological problems or the death a relative). The remained 60 women were interviewed by the author to fill out the DASS-21 and the SF-12 Quality of Life Scale and then, they were allocated to the intervention and control groups (30 participants apiece). All 30 women of intervention group completed the six counseling sessions. In this study, 4 women in the intervention group (one for reluctance to participate in the study, one for spouse’s dissatisfaction, and two for continuing treatment in Tehran) and 4 women in the control group (two for the impossibility of phone contact, one for a repeated pregnancy test and a positive result, and one for the possibility of separation from the spouse) left the study in the follow-up stage. Therefore, 26 women in the intervention and 26 women in the control group were followed up and analyzed in the post-intervention evaluation (Fig. 1).

To investigate the similarity between the groups in terms of socio-demographic profile, the chi-square, trend chi-square test, Fisher’s exact test, and the independent t-test were used. There was no significant difference between the intervention and control groups in the socio-demographic profile of participants (*P* > 0.05). The mean (SD: standard deviation) duration of infertility was 6.8 (3.1) and 6.8 (3.0) years in the intervention and control groups respectively. About 33% of participants in the intervention and 30% of them in the control groups had academic education and most participants in both groups were housewives (63% in the intervention group and 70% in the control group). About 67% of participants in the intervention group and 70% of them in the control group had no pregnancy experience. In addition, 67 and 60% of women in the intervention and control groups, respectively, reported sufficient income for living expenses. Marital satisfaction rate was 37 and 30% in the intervention and control groups, respectively (Table 1).

**Table 1:** Socio-demographic characteristics of participants in study groups

Characteristic	Counseling (***n =*** 26) Number (%)	Control (***n*** = 26) Number (%)	***P***-value
**Age (years)**^**a**^	(6.0)34.4	(7.4) 32.9	**0.179**^**c**^
**Husband’s age (years)**^**a**^	(6.9) 36.9	35.9 (7.9)	**0.455**^**c**^
**Duration of infertility (years)**^**a**^	6.8 (3.1)	6.8 (3.0)	**0.868**^**c**^
**Duration of treatment (years)**^**a**^	3.3 (1.7)	2.8 (1.4)	**0.912**^**c**^
**Type of infertility**			**0.784**^**b**^
Primitive	(66.7)20	21 (70.0)	
Secondary	10 (33.3)	9 (30.0)	
**IVF failure based on causes of infertility**			**0.445**^**b**^
**Male agent**			
First time	(23.3)7	(23.3)7	
Second time and more	(26.7)8	(26.7)8	
**Female agent**			
First time	(26.7)8	(26.7)8	
Second time and more	(23.3)7	(23.3)7	
**Education**			**0.621**^**d**^
Guidance	2 (6.7)	1 (3.3)	
High school	4 (13.3)	2 (6.7)	
Diploma	14 (46.7)	18 (60.0)	
University	10 (33.3)	9 (30.0)	
**Job**			**0.584**^**b**^
Housewife	19 (63.3)	21 (70.0)	
Employed	11 (36.7)	9 (30.0)	
**Spouse’s education**			**0.929**^**d**^
Illiterate	(3.3)1	(6.7)2	
Elementary	(10.0)3	(13.3)4	
Secondary school	(20.0)6	(13.3)4	
High school	(23.3)7	(20.0)6	
Diploma	(26.7)8	(26.7)8	
University	(16.7)5	(20.0)6	
**Spouse’s job**			**0.874**^**e**^
Jobless	3 (10.0)	2 (6.7)	
Employee	5 (16.7)	6 (20.0)	
Worker	11 (36.7)	10 (33.3)	
Shopkeeper	10 (33.3)	8 (26.7)	
Other	1 (3.3)	4 (13.3)	
**Number of children**			**1.000**^**d**^
0	(76.7)23	(73.3)22	
1	(13.3)4	(20.0)6	
2	(10.0)3	(6.7)2	
**Gravid**			**0.760**^**d**^
0	(66.7)20	(70.0)21	
1	(10.0)3	(10.0)3	
**≥ 2**	(23.3)7	(20.0)6	
**Number of deliveries**			**0.864**^**d**^
0	(70.0)21	(73.3)22	
1	(13.3)4	(13.3)4	
2	(16.7)5	(13.3)4	
**Number of abortions**			**1.000**^**d**^
0	(86.7)26	(93.3) 28	
1	(13.3)4	(3.3) 1	
2	(0.0)0	(3.3) 1	
**Number of stillbirths**			**0.232**^**d**^
0	(83.3)25	(93.3)28	
1	(16.7)5	(6.7)2	
**Having saved fetus**	(43.3)13	(56.7)17	**0.793**^**b**^
**Trying to conceive with a donated egg**	(6.7)2	(3.3)1	**0.684**^**e**^
**income level**			**0.460**^**d**^
Adequate	(0.0)0	(3.3)1	
Inadequate	(33.3)10	(36.7)11	
Relatively adequate	(66.7)20	(60.0)18	
**Marital life Satisfaction**			**0.399**^**d**^
**Satisfied**	(36.7)11	(30.0)9	
**Unsatisfied**	13 (43.3)	(40.0)12	
**Relatively satisfied**	(20.0)6	(30.0)9	
**Concerns about family relationships**			**0.519**^**b**^
**Yes**	(76.7)23	(83.3)25	

^a^Mean (SD)

^b^Chi-square test

^c^Chi-square test

^d^Independent T-test

^e^Fisher’s exact test

The mean (SD) total score of stress before the intervention was 16.6 (2.1) and 16.9 (1.7) in the intervention and control groups, respectively. The independent t-test indicated that there was no significant difference between the two groups before the intervention (*p* = 0.602). The mean (SD) total score of stress was 14.7 (2.8) and 16.7 (2.2) 1 week after the intervention and 15.2 (2.9) and 16.7 (2.2) 1 month after the intervention in the intervention and control groups, respectively. Based on the repeated measures ANOVA, the mean score of stress (after adjusting the baseline values) in the intervention group was significantly lower than that of the control group (adjusted mean difference = − 1.7, 95% confidence interval (CI): − 3.2 to − 0.3, *P* = 0.018) (Table 2).

**Table 2:** Comparison of mean stress scores in the study groups

Stress score (score 0 to 21)	Counseling group (***N =*** 26) Mean)SD)	Control group (***N*** = 26) Mean)SD)	MD (95%CI)	***p-***value
Before intervention	16.6 (2.1)	16.9 (1.7)	0.26(−0.75 to 1.2)	**0.602**^**a**^
One week after intervention	14.7 (2.8)	16.7 (2.2)	−1.7 (−3.2 to −0.3)	**0.018**^**b**^
One month after intervention	15.2 (2.9)	16.5 (2.3)		

^a^Independent t-test

^b^repeated measures ANOVA after adjusting the baseline stress value

Before intervention, the number of patients in the counseling and control groups was 30, and after the intervention in the counseling and control groups was 26

The mean (SD) total score of anxiety before the intervention was 15.7 (3.0) and 15.7 (2.8) in the intervention and control groups, respectively. The independent t-test showed that there was no significant difference between the two groups before the intervention (*p* = 0.965). The mean (SD) total score of anxiety was 13.3 (2.6) and 15.5 (2.9) 1 week after the intervention and 15.3 (2.8) and 15.5 (2.9) 1 month after intervention in the intervention and control groups, respectively. Based on the repeated measures ANOVA, there was no significant difference between the intervention and control groups in the mean score of anxiety after adjusting the baseline values (adjusted mean difference = − 1.1, 95% CI: − 2.6 to 0.4, *P* = 0.153) (Table 3).

**Table 3:** Comparison of the mean anxiety scores in the study groups

Anxiety Score (score 0 to 21)	Counseling group (***N*** = 26) Mean)SD)	Control group (***N =*** 26) Mean)SD)	MD (95%CI)	***p-***value
Before intervention	15.7 (3.0)	15.7 (2.8)	−0.0 (− 1.5 to 1.4)	**0.965**^**a**^
One week after intervention	13.3 (2.6)	15.5 (2.9)	−1.1 (−2.6 to 0.4)	**0.153**^**b**^
One month after intervention	15.3 (2.8)	15.7 (2.9)		

^a^Independent t-test

^b^repeated measures ANOVA after adjusting the baseline anxiety value

Before intervention, the number of patients in the counseling and control groups was 30, and after the intervention in the counseling and control groups was 26

The mean (SD) total score of depression before the intervention was 15.9 (3.0) and 16.0 (2.7) in the intervention and control groups, respectively. The independent t-test demonstrated that there was no significant difference between the two groups before the intervention (*p* = 0.965). The mean (SD) total score of depression was 12.2 (3.1) and 15.9 (2.7) 1 week after the intervention and 13.3 (2.9) and 15.9 (2.7) 1 month after intervention in the intervention and control groups, respectively. Based on the repeated measures ANOVA, after adjusting the baseline values, the mean score of depression in the intervention group was significantly lower than that of the control group (adjusted mean difference = − 3.1, 95% CI: − 4.7 to − 1.5, *P* < 0.001) (Table 4).

**Table 4:** Comparison of the mean depression scores in the study groups

Depression score (score 0 to 21)	Counseling group (***N =*** 26) Mean)SD)	Control group (***N =*** 26) Mean)SD)	MD (95%CI)	***p-***value
Before intervention	(3.0) 15.9	(2.7) 16.0	0.0(−1.4 to 1.5)	**0.965**^**a**^
One week after intervention	(3.1) 12.2	(2.7) 15.9		
One month after intervention	(2.9) 13.3	(2.7) 15.5	−3.1(−4.7 to −1.5)	**0.001 < 0**^**b**^

^a^Independent t-test

^b^repeated measures ANOVA after adjusting the baseline depression value

Before intervention, the number of patients in the counseling and control groups was 30, and after the intervention in the counseling and control groups was 26

The mean (SD) total score of Quality of Life before the intervention was 31.8 (3.7) and 32.1 (3.5) in the intervention and control groups, respectively. The independent t-test indicated that there was no significant difference between the two groups before intervention (*p* = 0.778). The mean (SD) total score of QoL was 40.3 (3.5) and 32.2 (3.5) 1 week after the intervention and 38.1 (3.1) and 32.2 (3.5) 1 month after the intervention in the intervention and control groups, respectively. Based on the repeated measures ANOVA, after adjusting the baseline values, the mean score of QoL in the intervention group was significantly lower than that of the control group (adjusted mean difference = 6.9, 95% CI: 5.1 to 8.8, *P* < 0.001) (Table 5).

**Table 5:** Comparison of the mean quality of life scores in the study groups

Quality of life score (score 0 to 21)	Counseling group (***N =*** 26) Mean)SD)	Control group (***N =*** 26) Mean)SD)	MD (95%CI)	***p-***value
Before intervention	(3.7) 31.8	(3.5) 32.1	0.2(−1.6 to 2.1)	**0.778**^**a**^
One week after intervention	(3.5) 40.3	(3.5) 32.2	6.9(5.1 to 8.8)	**0.001 > 0**^**b**^
One month after intervention	(3.1) 38.1	(3.5) 32.4		

^a^Independent t-test

^b^repeated measures ANOVA after adjusting the baseline quality of life value

Before intervention, the number of patients in the counseling and control groups was 30, and after the intervention in the counseling and control groups was 26

There were not any reported adverse events in this study.

## Discussion

Most psychologists today believe that hope is effective in the treatment of most adverse consequences in humans [35]. Previous studies have confirmed and emphasized the significance of hope-oriented group counseling in reducing physical and mental complications caused by various events [36–38]. It is especially important in the prevention of psychological traumas in women and has been widely investigated [39, 40]. Most studies suggest that hope therapy based on positive psychological approaches highlights people’s strong points, instead of focusing on their weaknesses [36, 37, 41].

Given that interventional studies are the best for producing evidence with the highest level among initial studies;this study aimed to determine the effects of hope-oriented group counseling on the mental health of women with failed IVF cycles. The findings suggested that hope-oriented group counseling significantly reduced stress and depression scores and increased the quality of life score.

The study results showed that the mean score of stress reduced significantly in the intervention group compared to the control group 1 week and 1 month after the intervention. The authors found no contradictory findings in this regard in the literature. This finding is consistent with the results of Roodsari et al. [24], Rabi’i Pour et al. [42], Kim et al. [43], and Karaca et al. [44] who introduced and suggested group counseling as an effective factor in stress management in women with failed IVF cycles visiting infertility clinics. Lopez and Snyder [45, 46] argue that hope, as a psychological construct, consists of two concepts, including the ability to design pathways toward intended goals despite existing barriers and the necessary factor or motivation to use these pathways. Humans are both positive and negative and it is time to pay more attention to their abilities and positive aspects rather than their vulnerabilities. Hope is at the center of the positive half, and the main advantage of hope therapy, compared to other psychotherapeutic treatments, is that it creates hopeful thoughts towards life to deal with the problems and their negative effects on various aspects of life. Having hopeful thoughts and sufficient resources to achieve goals improve psychological well-being as they enhance the meaningfulness of life, life satisfaction, QoL, etc. According to Retnowati et al. [47], Hopefulness is an effective coping strategy in the face of stress and tension. Hopeful people have more pathways and factors to reach their goals and can maintain their motivation and use alternative pathways to deal with problems. Hopeless people, by contrast, have fewer pathways and factors and may lose their motivation and experience negative emotions in the face of problems and barriers. In other words, pathways or factors are personal beliefs that make individuals feel that they can try and endure different paths to achieve their goals, which is possible only in the light of hope. Teaching hope or hope therapy provide individuals with more pathways and factors and leads to psychological well-being by increasing their achievements, self-esteem, self-efficacy, etc.

The findings also showed that the mean score of anxiety in the intervention group was lower than that of the control group 1 week and 1 month after the intervention. However, there was no significant difference between the two groups in this regard. This is consistent with the results of Klerk et al. [48] who found no significant difference between the 265 couples visiting an infertility clinic in the Netherlands in the mean score of anxiety. In this study, women in the intervention group received hope-oriented group counseling before, during, and after the first IVF cycle. The authors concluded that low response to treatment indicates that couples need no group counseling and psychotherapeutic treatment in the first IVF cycle. By contrast, this result was not consistent with the findings of Zarghami et al. [49], Yeylagh Beigi et al. [8], Likhachov et al. [50], and Joelsson et al. [51]. These contradictory results can be attributed to the difference in sample size and measurement tools. The authors suggest that similar studies be conducted on larger samples in order to achieve more valid, accurate, reliable, and generalizable results.

The study results suggested that the mean score of depression in the intervention group 1 week and 1 month after the intervention was significantly lower compared to the control group (*P* < 0.001). This is consistent with the previous studies where behavioral-cognitive interventions and group psychological therapies were reported efficient in reducing depression in infertile women [51–55]. To explain this finding according to Janfada et al. [56], it can be stated that hope therapy increases hope in individuals as a positive force that boosts motivation, goal progression, and adaptation, and the power to maintain and boost energy in people is part of the nature of hope. Hopeful people may have stronger stimuli and more energy to pursue their goals, depending on their motivation to actively engage in the problem-solving process and take behaviors that lead to development and maturity. As an intervention, hope therapy increases people’s ability to cope with stressful and challenging situations and diseases. This type of treatment basically increases adaptation, optimism, assertiveness, and self-confidence in people. Therefore, they find stressful and challenging situations controllable and manageable and have optimistic attitudes that increase their resilience and ability to cope with difficult situations and save them from depression.

The study findings also demonstrated that the mean score of quality of life in the intervention group was significantly higher compared to the control group, indicating the effective and efficient role of interventions in improving QoL in women with failed IVF cycles. No contradictory result was found in the literature review, This finding is consistent with the results of Logiudice et al. [55], Wu et al. [54], and Hosseini et al. [57]. To explain this, hope is the possibility of a desirable event or behavior in the future. It is an important factor in the course of individual and group counseling and predicts positive outcomes in the future general health of patients. Hope can increase the level of self-care and QoL and promote general health. Hope therapy aims to promote hope and teach new and appropriate behaviors to deal with fertility problems, creates a sense of dominance and empowerment in promoting mental health of individuals, and increases their hope. Hopeful individuals enjoy more positive thoughts, self-esteem, and self-confidence compared to those who are less hopeful. More hopeful people express their emotions more energetically and present themselves in a more positive way. It also seems that the acquisition and application of coping skills based on hope therapy strengthens the self-efficacy in such people. On the other hand, familiarization of clients with life skills in difficult situations helps them to think about all aspects of situations, respond to pressures and stresses imposed by infertility more appropriately, and better accept and act on the solutions offered [58, 59]. QoL is composed of different dimensions and is affected by several factors. However, the desirable effects of combination therapy based on acceptance, commitment, and hope therapy indicate the value and effectiveness of this intervention in improving QoL of women with failed IVF cycles [60].

### Limitations and strengths

In this study, all answers provided by the participants were assumed to be correct, and it was beyond the researcher’s ability to ensure the answers were correct. Since this was a small study in a single center and all participants were literate, the study findings should be generalized very cautiously. All principles of clinical trials, including random allocation and concealment of allocation, were observed in this study. Moreover, the authors filled out the questionnaires to reduce the likelihood of incomplete, unanswered, and incorrect answers. In addition, to communicate more effectively with the participants, their vernacular language was used in the counseling sessions.

## Conclusion

The study results indicated that hope-oriented group counseling significantly reduced the mean scores of stress and depression and increased the mean score of quality of life. Since infertile women require mental and psychological support, in addition to physical support, to improve fertility outcomes, mental health, and quality of life, health care providers and infertility treatment staff can offer this counseling method along with reproductive care to clients with a history of infertility.

## Data Availability

Data and materials of this study are available from the corresponding author upon reasonable request.

## Abbreviations

IVF: In Vitro Fertilization
QoL: Quality of Life
DASS: Depression Anxiety Stress Scale
ICC: Intra-Correlation Coefficient
